# An innovative method to prevent infection when measuring the arterial blood gas SpO2 saturation

**DOI:** 10.3205/dgkh000473

**Published:** 2024-04-17

**Authors:** Seda Şahan, Sevil Güler, Eliz Geçtan, Hakan Aygün

**Affiliations:** 1 Izmir Bakircay University, Health Sciences Faculty, Nursing Department, İzmir, Turkey; 2 Erciyes University, Health Sciences Faculty, Nursing Department, Kayseri, Turkey; 3 İzmir Bakircay University Cigli Education and Research Hospital, Anaesthesia, İzmir,Turkey

**Keywords:** intra-arterial blood gas measurement, oxygen saturation, pulse oximeter, cross infection, glove

## Abstract

**Background:**

Patients are hospitalized for extended periods, particularly in intensive care units (ICUs). As a result, the saturation probe (pulse oximeter) remains attached for an extended period and microorganisms can grow in the wet environment. If the pulse oximeters are not reprocessed, cross-infection may occur. The literature contains several studies in which gloves were used for the measurement while various SpO_2_ (peripheral arterial oxygen saturation) measurements were compared with each other. However, such comparisons have yet to be made with the results of arterial blood gas SpO_2_ measurements by pulse oximeter, considered as the gold standard. The present study aimed to compare arterial blood gas values with the fingertip saturation measurement performed by having adult patients wear gloves of different colors, one after the other, on their fingers and determining the effect of the differently colored gloves (transparent, white, black, light blue) on saturation values.

**Methods:**

The study was conducted on 54 patients in an ICU. Intra-arterial blood gas SpO_2_ results were measured. Oxygen saturation was measured while the patient 1. did not wear gloves and 2. sequentially wore a series of gloves of different colors. Paired t-test, correlation analysis, and Bland Altman charts were used to evaluate the results.

**Results:**

The mean SpO_2_% value of the participants’ intra-arterial blood gas measurements was 97.76±2.04. The mean SpO_2_% value obtained from the measurements of the fingers with a transparent glove was 0.43 points lower than the mean SpO_2_% value of the intra-arterial blood gas measurements (t=0.986, p=0.61). The mean SpO_2_% value obtained from the measurements of the fingers with a white glove was 0.93 points lower than the mean SpO_2_% value of the intra-arterial blood gas measurements (t=1.157, p=0.093).

**Conclusion:**

Of the measurements performed with a glove, the mean SpO_2_% value obtained from the measurements of the fingers with a transparent glove was more consistent with the mean SpO_2_% value of the intra-arterial blood gas measurements than measurement of the fingers without a glove.

## Zusammenfassung

**Hintergrund:**

Patienten werden über längere Zeiträume stationär behandelt, insbesondere auf Intensivstation und Mikroorganismen können sich in der feuchten Kammer vermehren. Werden die Sättigungssonden nicht aufbereitet, kann es zu Kreuzinfektionen kommen. In der Literatur wurden mehrere Studien durchgeführt, in denen Handschuhe zur Messung verwendet wurden, wobei verschiedene SpO_2_-Messungen miteinander verglichen wurden. Solche Vergleiche mit den Ergebnissen arterieller Blutgas-SpO_2_-Messungen, die als Goldstandard gelten, stehen jedoch aus. Ziel der vorliegenden Studie war es, arterielle Blutgaswerte mit der Sättigungsmessung an den Fingerspitzen bei der erwachsene Patienten zu vergleichen, die transparente Handschuhe unterschiedlicher Farbe an den Fingern trugen, um die Auswirkung von Handschuhen auf die Sättigungswerte zu bestimmen.

**Methode:**

Die Studie wurde an 54 Patienten auf einer Intensivstation durchgeführt. Die intraarteriellen Blutgas-SpO_2_-Ergebnisse der Patienten wurden gemessen. Die Sauerstoffsättigung wurde gemessen, wenn der Patient keine Handschuhe bzw. einen andersfarbigen Handschuh trug. Bei der Analyse der Studie wurden der gepaarte T-Test, die Korrelationsanalyse und Bland-Altman-Diagramme verwendet.

**Ergebnisse:**

Der mittlere SpO_2_%-Wert der intraarteriellen Blutgasmessungen betrug 97,76±2,04. Der mittlere SpO_2_%-Wert aus den Messungen der Finger mit einem transparenten Handschuh war 0,43 Punkte niedriger als der mittlere SpO_2_%-Wert der intraarteriellen Blutgasmessungen (t=0,986, p=0,61). Der mittlere SpO_2_%-Wert aus den Messungen der Finger mit einem weißen Handschuh war 0,93 Punkte niedriger als der mittlere SpO_2_%-Wert der intraarteriellen Blutgasmessungen (t=1,157, p=0,093).

**Schlussfolgerung:**

Bei den mit Handschuhen durchgeführten Messungen stimmte der mittlere SpO_2_%-Wert, der aus den Messungen an den Fingern mit einem transparenten Handschuh erhalten wurde, besser mit dem mittleren SpO_2_%-Wert der intraarteriellen Blutgasmessungen überein als bei Messung am Finger ohne Handschuh.

## Introduction

The pulse oximeter is a non-invasive device that measures the oxygen saturation in the blood by emitting specific wavelengths of light through the tissue [1]. Although pulse oximeter probes can be placed in different body parts, measurement is most often performed by attaching them to the fingers or toes [2]. 

Pulse oximeters are one of the most commonly used devices in healthcare services. They are used to monitor peripheral blood oxygen saturation [3]. In the hospital setting, pulse oximeter probes are usually connected to a monitor via cables. However, there are also pulse oximeters with a small monitor screen on the device which can be used easily by patients without being connected to a monitor [4]. Although pulse oximeters are easy-to-use devices, they pose a risk of infection for patients [5]. In particular, high-level disinfection procedures should be applied to probes contaminated with body fluids [6]. Disinfection of probes cannot be regularly achieved in hospital units because patient circulation is high, and time needed for disinfection is inadequate [7]. 

When the pulse oximeter is worn by a patient for a long time, sebum accumulates inside the probe, which creates a suitable environment for the growth of microorganisms [8], which has been shown in some studies [9]. In such studies, it has also been determined that pulse oximeters play a role in the transmission of hospital-acquired infection (HAI) [8]. In particular, attaching probes having been worn by patients with infectious diseases to other patients without disinfection causes cross-infections and contamination [10]. In the literature, it has been indicated that the rate of microorganism contamination of pulse oximeters ranges between 66% and 80% [7], [11].

An option to prevent pulse-oximeter induced cross-infection and contamination is to use disposable probes. As stated in the literature, single-use pulse oximeters are being developed [12], [13]. However, arguments against their use are the higher costs [13] and the lack of sustainability [14].

Due to the growth of microorganisms on the inner surfaces of the probes, precautions should be taken in this regard. For instance, gloves are easily accessible materials frequently used in hospitals. In the literature, several studies exist comparing different gloves worn during various SpO_2_ measurements. However, such comparisons have yet to be made with the results of arterial blood gas SpO_2_ measurements, considered as the gold standard. Therefore, the purpose of the present study was to compare arterial blood gas values taken from fingertip saturation measurements performed while adult patients sequentially wore gloves of different colors on their fingers, and to determine the effect of gloves on saturation values. Thus, by using gloves which are the most accessible material for patients and healthcare personnel, the main aim was to prevent probe-induced infections. 

## Methods

### Setting

The study was performed in a training and research hospital in Izmir, Turkey. The population of the study consisted of patients who were hospitalized in the three General Intensive Care Units of the hospital between November and December 2022. 

### Sample size calculation

To determine the sample size of the study, G*power analysis (G*Power 3.1.9) was performed by taking into account the sample sizes of similar studies in the literature [5], [15]. According to the results of the power analysis thus performed, the effect size was calculated as 0.12, based on the benchmark suggested by Cohen for the medium effect size (0.15), considering that there might be a 20% deviation (alpha value: 0.05, confidence interval: 95%). According to Cohen’s f^2^, 0.02 indicates a low effect size, 0.15 a medium effect size, and 0.35 a large effect size. Therefore, 54 patients were included to achieve 95% power.

### Inclusion criteria

Had no nail polish, wounds, ulcers, burns on fingers, no amputated fingers, had arterial blood gas measured, volunteered to participate in the study.

### Data collection process

First, the patients or their relatives were interviewed and informed about the study, and their informed consent was obtained. Second, the data on the descriptive characteristics of the patients who gave their informed consent were recorded in the Patient Information Form. Third, the patients’ oxygen saturation was measured with a bedside pulse oximeter placed on their fingers. 

### Measuring procedure

**Figure 1:**
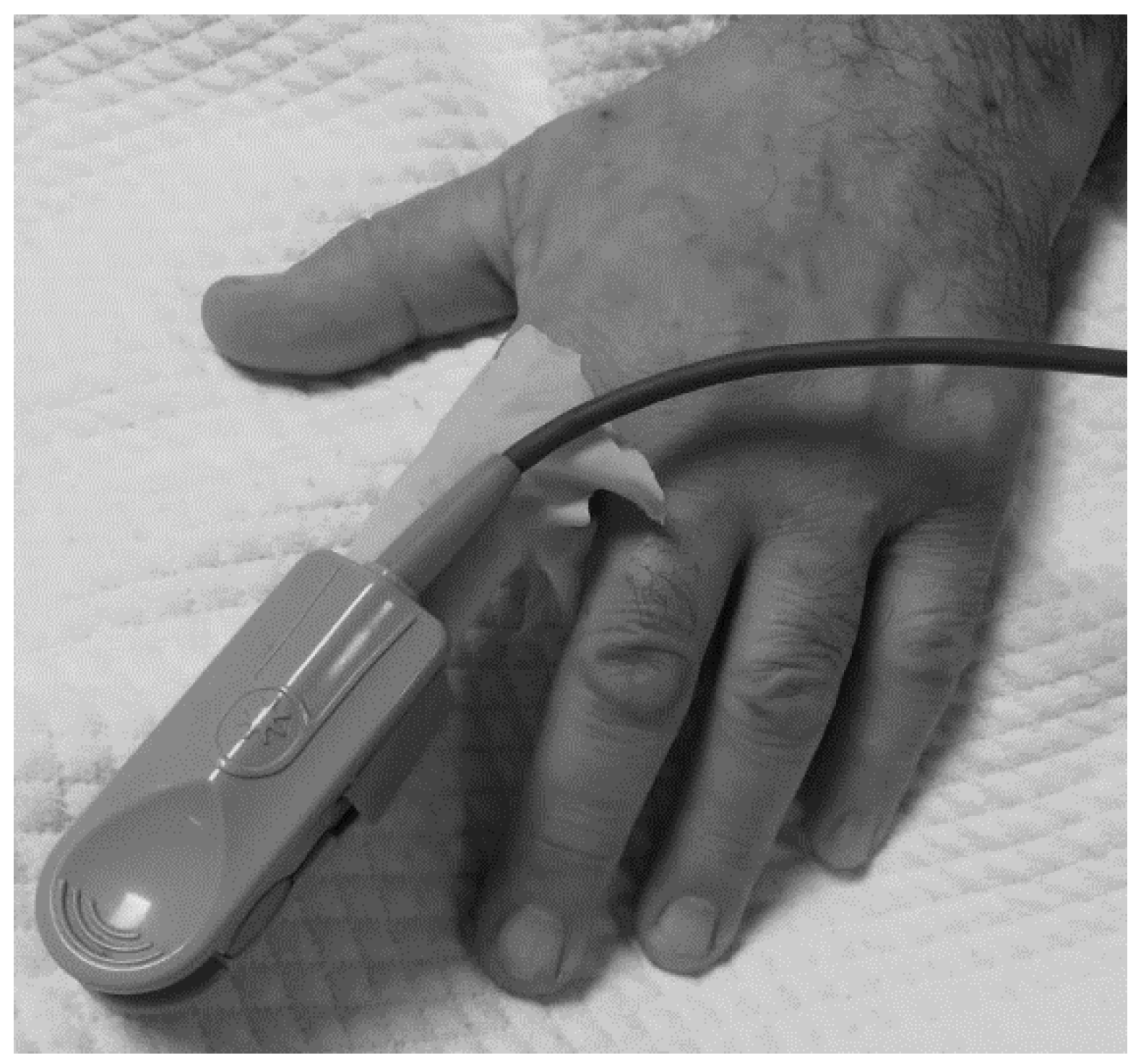
Photograph of a patient’s gloved finger in the oximeter

The measurements were performed as follows:

- *First measurement*: Patients whose intra-arterial blood gas was measured were determined. SpO_2_ measurements on the finger were started as soon as the blood gas measurement process started. Measurements made with the pulse oximeter and intra-arterial blood gas measurements were performed simultaneously.
- *Second measurement*: While the patient was not wearing gloves, the pulse oximeter light source was placed on the outer surface of the finger (on the nail). SpO_2_ was read after waiting an average of 30 seconds.
- *Third measurement*: While the patient was wearing a transparent glove, the pulse oximeter light source was placed on the outer surface of the finger (on the nail). SpO_2_ was read after waiting an average of 30 seconds.
- *Fourth measurement*: While the patient was wearing a white glove, the pulse oximeter light source was placed on the outer surface of the finger (on the nail). SpO_2_ was read after waiting an average of 30 seconds (Figure 1 (Fig. 1)).
- *Fifth measure*ment: While the patient was wearing a black glove, the pulse oximeter light source was placed on the outer surface of the finger (on the nail). SpO_2_ was read after waiting an average of 30 seconds.
- *Sixth measurement*: While the patient was wearing a light blue glove, the pulse oximeter light source was placed on the outer surface of the finger (on the nail). SpO_2_ was read after waiting an average of 30 seconds.

The measurements were performed consecutively without any intervals in between. Measurements were made on the index finger while the patient was in the supine position. Measurements made with the pulse oximeter and intra-arterial blood gas measurements were performed simultaneously. While the patients’ blood gas was measured, they wore gloves of different colors. Because there were no intervals between the measurements, the variation of oxygen saturation over time was minimized. Oxygen saturation measurements were performed by one (the same) researcher using a GE-brand bedside pulse oximeter.

### Analysis of the data

The data were analyzed using the Statistical Package for Social Sciences (SPSS) program. In the analysis, numbers (n) and percentages were recorded, the results were statistically analyzed using the paired t-test and correlation analysis. The results obtained from the analysis of the data were evaluated at a significance level of p<0.05 and a confidence interval of 95%.

### Ethical issues

The study was approved by the Ethics Committee of Izmir Bakirçay University Non-Interventional Clinical Research (No: 738-718). Of the patients to be included in the study, those who were conscious were interviewed face-to-face. If the patient was unconscious or semi-conscious, their relatives were interviewed. During the interviews, the patients or their relatives were informed about the study.

## Results

The mean age of the participants was 60.8±13.4% years. Of the participants, 48.1% were women; 83.3% had a chronic disease. The mean SpO_2_% value of the measurements of the intra-arterial blood gas of the participants was 97.76±2.04. The SpO_2_% value was 97.42±1.57 when the measurement was made on a finger without a glove, 97.33±1.71 when the patient wore a transparent glove, 96.83±1.69 when the patient wore a white glove, 95.20±3.19 when the patient wore a light blue glove, and 91.12±3.74 when the patient wore a black glove (Table 1 (Tab. 1)).

The comparison of the mean SpO_2_% of the intra-arterial blood gas (IABG) measurements and the mean SpO_2_% values obtained from the measurements of the fingers without a glove or with gloves of different colors revealed the following: The mean SpO_2_% value obtained from gloveless fingers was 0.34 points lower than the mean SpO_2_% value of the IABG (t=1.918, p=0.83). The mean SpO_2_% value obtained from the fingers wearing a transparent glove was 0.43 points lower than the mean SpO_2_% value of the IABG measurements (t=0.986, p=0.61). The mean SpO_2_% value of the fingers wearing a white glove was 0.93 points lower than the mean SpO_2_% value of the IABG measurements (t=1.157, p=0.093). The mean SpO_2_% value obtained from fingers wearing a light blue glove was 2.56 points lower than the mean SpO_2_% value of the IABG measurements (t=5.237, p=0.023). The mean SpO_2_% value obtained from fingers with a black glove was 6.63 points lower than the mean SpO_2_% value of the IABG measurements (t=8.716, p=0.00). Of the measurements performed with gloves, the mean SpO_2_% value with a transparent glove was more consistent with the mean SpO_2_% value of the IABG measurements (Table 2 (Tab. 2)).

The comparison of the mean SpO_2_% value obtained from the fingers without a glove and the mean SpO_2_% value obtained from the fingers with gloves of different colors revealed the following: The mean SpO_2_% value obtained from the fingers with a transparent glove was 0.09 points lower than the mean SpO_2_% value obtained from the gloveless fingers (t=0.489, p=0.62). The mean SpO_2_% value obtained from the fingers with a white glove was 0.59 points lower than the mean SpO_2_% value obtained from the gloveless fingers (t=0.614, p=0.12). The mean SpO_2_% value obtained from the fingers with a light blue glove was 2.22 points lower than the mean SpO_2_% value obtained from the fingers with a glove (t=4.465, p=0.00). The mean SpO_2_% value obtained from the fingers with a black glove was 6.29 points lower than the mean SpO_2_% value obtained from the fingers without a glove (t=5.577, p=0.00). The mean SpO_2_% value obtained from fingers with a transparent glove was more consistent with the mean SpO_2_% value obtained from gloveless fingers (Table 3 (Tab. 3)).

Pearson’s correlation was used to test the relationship between the mean SpO_2_% value of the IABG measurements and the mean SpO_2_% values obtained from gloveless fingers or with gloves of different colors. There was a positive significant correlation between the mean SpO_2_% value of the IABG measurements and the mean SpO_2_% values obtained from fingers without a glove (r=0.813, p=0.00). There was a positive significant correlation between the mean SpO_2_% value of the IABG measurements and the mean SpO_2_% values obtained from fingers with a transparent glove (r=0.937, p=0.001). There was a positive significant correlation between the mean SpO_2_% value of the IABG measurements and the mean SpO_2_% values obtained from fingers with a white glove (r=0.770, p=0.00). There was no correlation between the mean SpO_2_% value of the IABG measurements and the mean SpO_2_% values obtained from fingers wearing a light blue glove (r=0.111, p=0.42). No correlation was found between the mean SpO_2_% value of the IABG measurements and the mean SpO_2_% values obtained from fingers wearing a black glove (r=0.229, p=0.095) (Table 4 (Tab. 4)).

## Discussion

Pulse oximeters are among the most frequently used devices in healthcare institutions. Peripheral blood oxygen saturation is monitored using these devices [5], [16], and pulse oximeters are the first device used for early detection of the decrease in oxygen saturation [17], [18]. A pulse oximeter worn for a long time can increase sebum production, which creates a suitable environment for the growth of microorganisms. Contamination of the inner surface of the oximeter can also hinder the activity of disinfectants [11]. One study determined that growth of pathogenic microorganisms in 68% of contaminated pulse oximeter probes [11]. Using pulse oximeters in more than one patient without disinfecting them can cause cross-infections [10], [19]. The World Health Organization states that such saturation probes should be wiped with disinfectants [20]. The Center for Disease Prevention and Control (CDC) considers saturation devices (such as the pulse oximeter) as non-critical equipment and recommends low-level disinfection, i.e., several times a week, before and after patient contact [21]. Even in contaminated pulse oximeters, once disinfected, “neglected reservoirs” can form due to areas that are difficult to access, regardless of the product’s commercial brand. In addition, some environmental conditions, such as high temperature, can keep the contamination level high. In contrast, in the disinfection of non-critical environmental surfaces and equipment in patient care, the Centers for Disease Control and Prevention do not recommend the use of liquid chemical sterilizing agents or disinfectants such as glutaraldehyde, peracetic acid and the antiseptics chlorhexidine and iodophors. It also advises against the use of phenolics, with their high toxicity [22]. This recommendation needs to be adapted to the disinfection of finger oximeters. According to the Centers for Disease Control and Prevention, the inappropriate use of some of these products poses risks to health professionals, especially when used too frequently, and recommends caution in mixing substances for disinfection [22]. The presence of sebum reduces the cleaning efficacy of some commercially available wipes for some select microbes. One study found that 70% isopropanol specified for disinfecting oximetry probes significantly mechanically reduced spores but was not effective against them [8]. To avoid permanent damage, use excessive amounts of liquids to clean or disinfect the device is not advised [20]. The desired effect could not be achieved with disinfection by wiping. For this reason, this study was conducted to create a more reliable method than disinfection to prevent cross-contamination in cleaning pulse oximeters.

Therefore, in order to prevent cross-infections between patients, we developed an innovative approach for the use of pulse oximeters in clinics and at home without purchasing new devices. We measured the saturation values by having patients wear gloves of different colors on their fingers and compared the results with the gold-standard arterial blood gas.

Mondal et al. [5], who took saturation measurements using plastic bags of different colors covering the probe and the finger, reported that the saturation results for the white, yellow, transparent, green and red bags were similar, but those for the black bag were different. However, they did not compare their results with the arterial blood-gas gold standard. In our study, the mean SpO_2_% value obtained from the fingers without a glove was 0.34 points lower than the mean SpO_2_% value of the IABG measurements. This value was more consistent with the mean SpO_2_% value of the IABG measurements. 

Of the measurements performed with a glove, the mean SpO_2_% value obtained from the fingers wearing a transparent glove was more consistent with the mean SpO_2_% value of the IABG measurements because the glove was transparent and colorless, which did not prevent the penetration of infrared rays from the probe into the fingernail bed.

Similarly, the mean SpO_2_% value obtained from the fingers with a white glove was 0.43 points lower than the mean SpO_2_% value of the IABG measurements, which indicated that the difference was not significant. Yek et al. [23] investigated the effect of nail-polish colors on saturation measurements. According to the results of their study, white nail polish did not affect the saturation result and led to a result similar to the saturation value measured from the finger without nail polish. According to the results of our study, the mean SpO_2_ value obtained from a white-gloved finger were more consistent with the mean SpO_2_ value of the IABG measurements; thus, we can conclude that in the clinic, SpO_2_ measurements can be made on a finger with a white glove.

In our study, the mean SpO_2_% values obtained from the measurements made on fingers with a light blue or black glove were significantly different form the mean SpO_2_% value of the IABG measurements. Perez et al. [24] investigated the effect of using blue gloves on the SpO_2_ value, finding that there was no clinically significant difference between the results of the SpO_2_ measurements made on gloved fingers and the results of the SpO_2_% measurements made without gloves.

Our literature search revealed few studies in which the effect of wearing a glove on the finger on the results of SpO_2_ measurements was investigated, but that there were various studies in which the effect of nail polish in different colors on the results of SpO_2_ measurements was investigated. In two studies, it was stated that the results of SpO_2_ measurements performed on a finger with black nail polish were significantly different from those performed on a finger without nail polish [25], [26]. Similarly, Yönt et al. [27] stated that dark (black, blue) nail polishes adversely affected SpO_2_ measurements and led to false results. Haq et al. [28] obtained similar results indicating that dark nail polish affected SpO_2_ levels. Based on these results, we can conclude that the black glove lowered SpO_2_ values because it more strongly absorbs light wavelengths. Thus, we recommend that when SpO_2_ is measured with a pulse oximeter in the clinic, black gloves should not be used to prevent infection transmission.

Reprocessing of pulse oximeters is difficult because all surfaces must be reached for cleaning and subsequent disinfection, because the internal surfaces of pulse oximeter probes may serve as hot spots for an array of pathogens. The literature clearly states that microbial contamination is detected during SpO_2_ examination using a pulse oximeter. A simple and safe alternative is to wear gloves on the hand to be measured. 

In the literature, there are studies in which SpO_2_ measurements were made using gloves or plastic bags. However, the results of these measurements were compared with the SpO_2_ results obtained from the bare fingers of the patients. In our study, to determine the effect of wearing a glove on the finger on the results of SpO_2_ measurements and to strengthen our results, we compared the results of SpO_2_ measurements made with a pulse oximeter with the results of arterial blood gas SpO_2_ measurements, which is considered as the gold standard. The results of the present study thus make a valuable contribution to the literature thanks to the arterial blood gas comparisons.

## Conclusion

To prevent cross-infections and hospital-acquired infections, it is recommended to have patients wear gloves on their fingers, since they can be easily accessed in the clinic. Our study results indicate that SpO_2_ values obtained from the measurements of fingers wearing transparent or white gloves were more consistent with SpO_2_ values obtained from intra-arterial blood gas measurements. We recommend that healthcare professionals implement our method because of the ready availability of gloves in clinics.

In our study, we used gloves of different colors, because these colors are the ones most frequently used ones in our country. However, in other countries, gloves with different colors may be available. Therefore, we recommend that in studies to be conducted in the future, researchers should use gloves whose colors are different from the colors of gloves used in our study. 

### Limitation of the study

The literature does not contain enough studies in which gloves were used to measure SpO_2_ with a pulse oximeter; therefore, we compared our results with the results of studies in which nail polishes of different colors were used, a possible limitation.

## Notes

### Competing interests

The authors declare that they have no competing interests.

### Funding source 

None.

### Consent for publication 

All authors reviewed and approved the publication of this manuscript.

### Authors’ ORCID

- Seda Sahan: 0000-0003-4071-2742
- Sevil Güler: 0000-0002-1707-7333
- Eliz Geçtan: 0000-0001-5410-2836
- Hakan Aygün: 0000-0002-6152-0857

## Figures and Tables

**Table 1:**
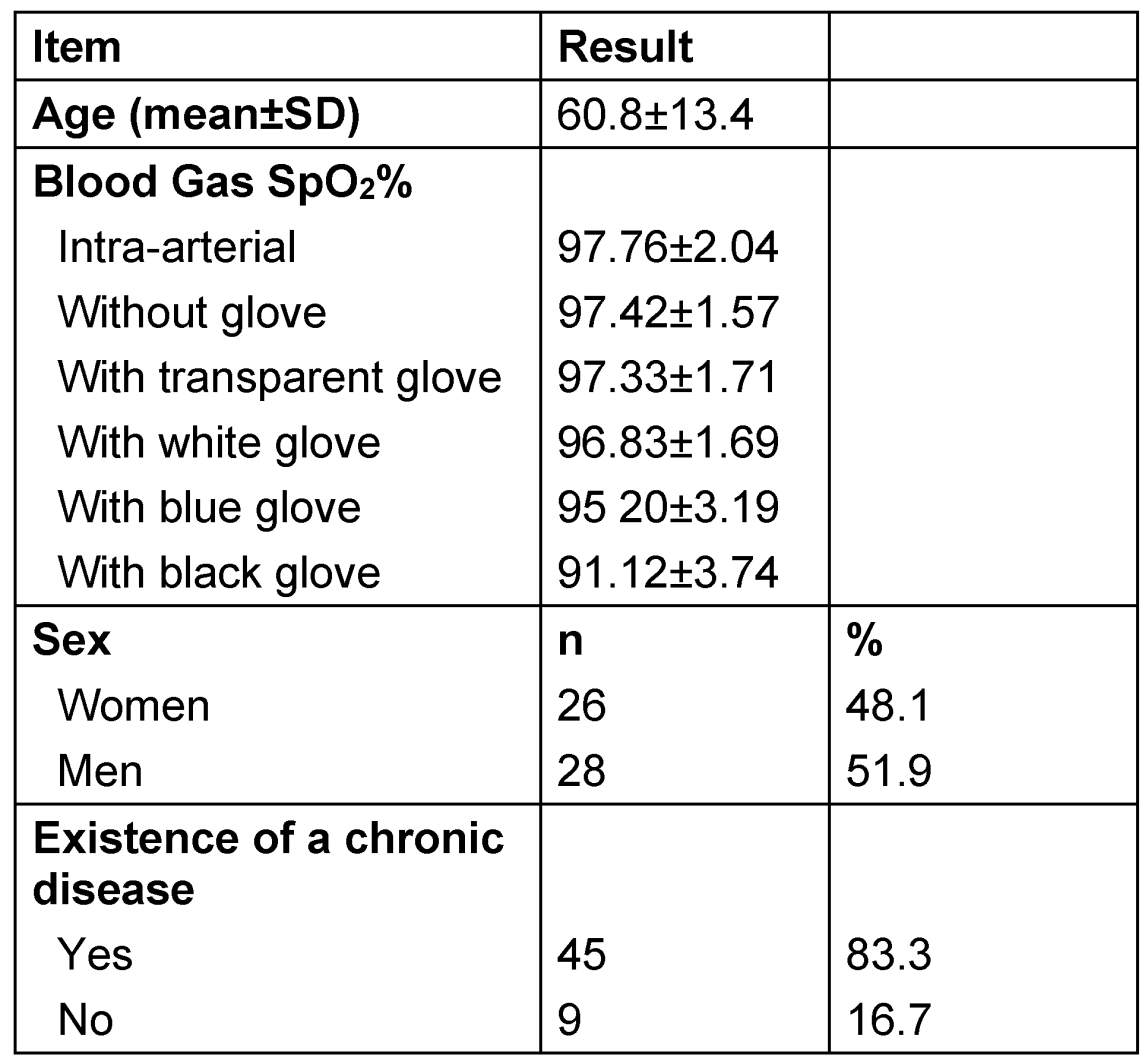
The participants’ characteristics

**Table 2:**
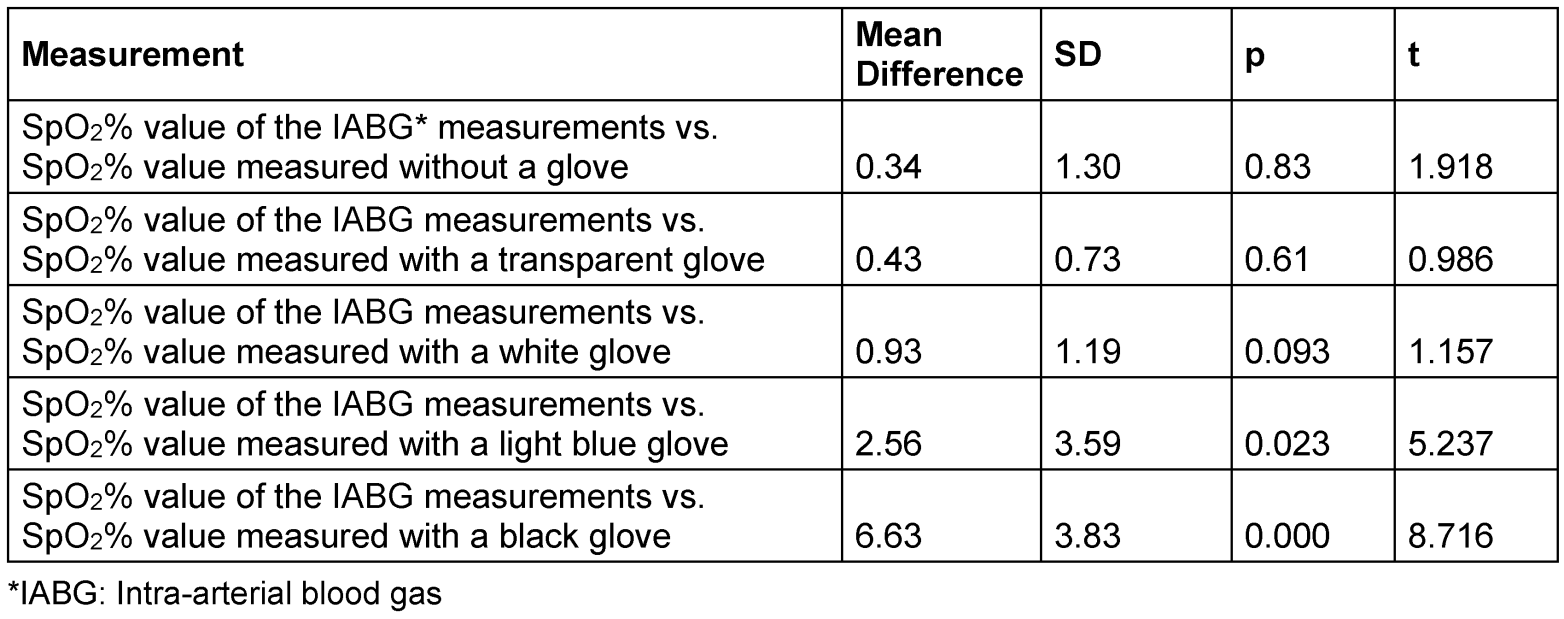
Comparison of mean IABG and SpO_2_% parameters

**Table 3:**
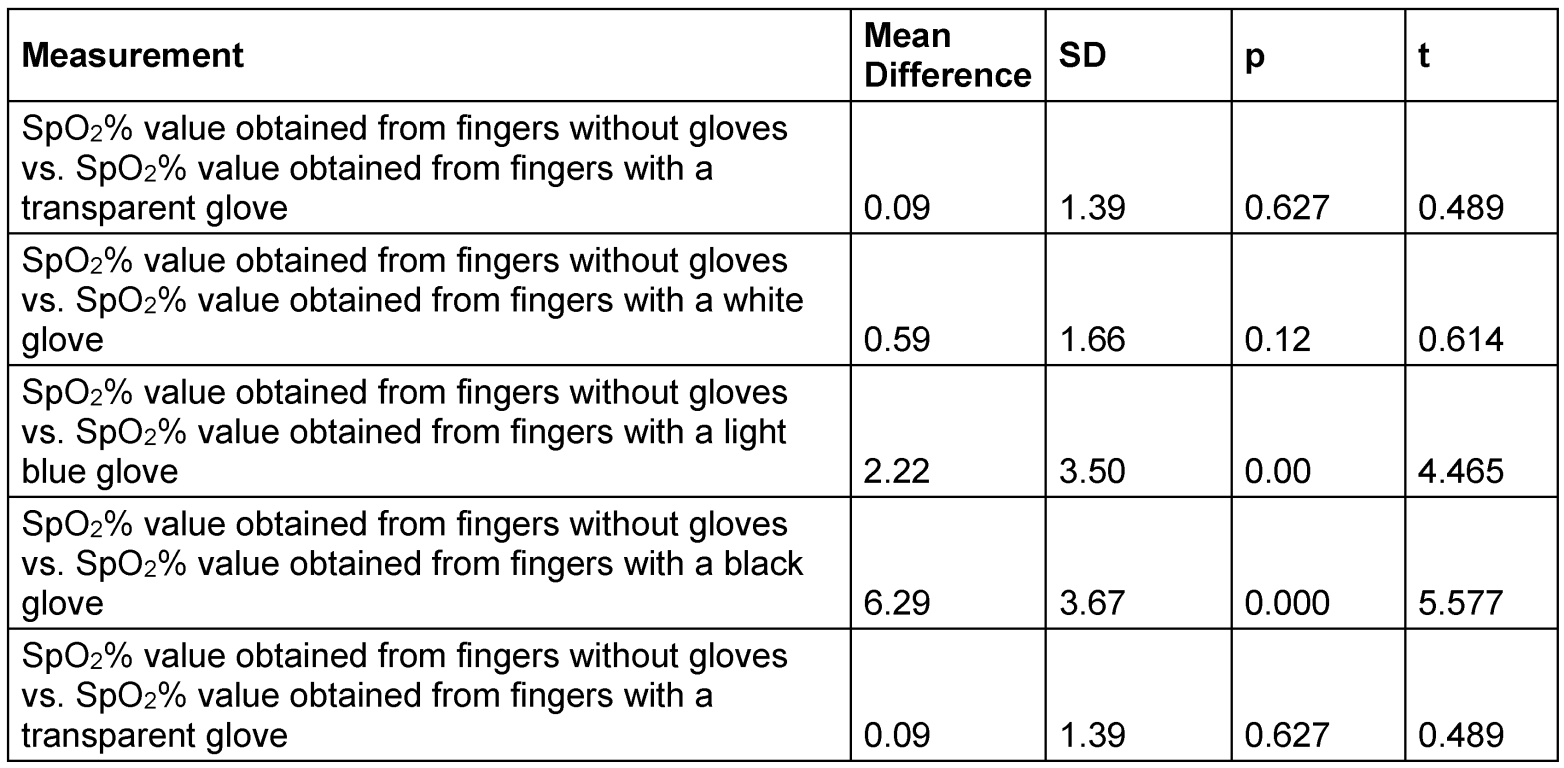
Comparison of mean SpO_2_% value obtained from fingers without a glove and mean SpO_2_% value obtained from t fingers with gloves of different colors

**Table 4:**
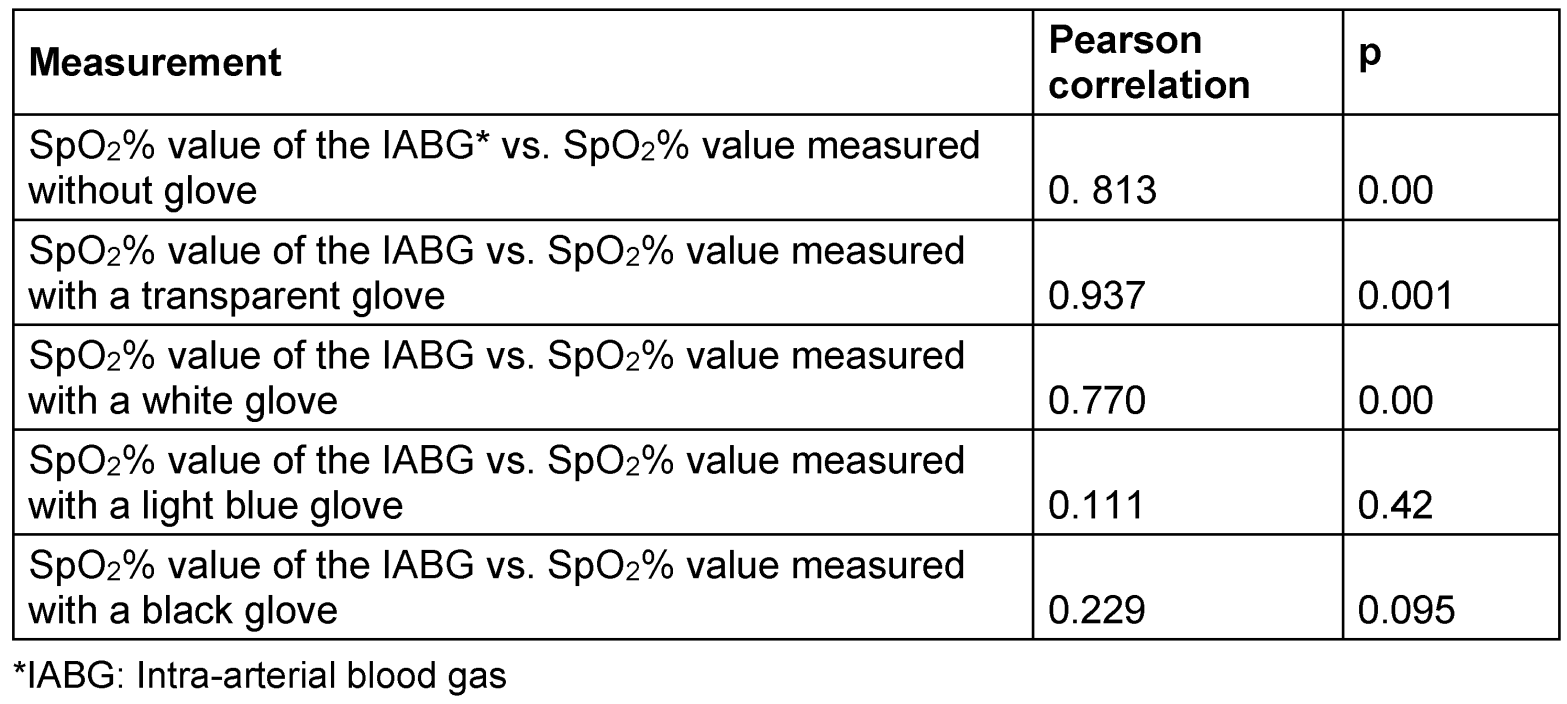
Comparison of correlation/SpO_2_% parameters
